# The Implication of Pathway Turn and Task Condition on Gait Quantified Using SmartWalk: Changes With Age and Parkinson’s Disease With Relevance to Postural Strategy and Risk of Fall

**DOI:** 10.3389/fnins.2022.804397

**Published:** 2022-04-29

**Authors:** Priya Pallavi, Neeti Jariwala, Niravkumar Patel, Manasi Kanetkar, Shraddha Diwan, Uttama Lahiri

**Affiliations:** ^1^Department of Electrical Engineering, Indian Institute of Technology Gandhinagar, Gandhinagar, India; ^2^SBB College of Physiotherapy, Ahmedabad, India; ^3^Design and Innovation Centre, Indian Institute of Technology Gandhinagar, Gandhinagar, India

**Keywords:** age, Parkinson’s, gait, posture, turnings, errors

## Abstract

One’s gait can be affected by aging, pathway with turns, task demands, etc., causing changes in gait-related indices and knee flexion (influencing posture). Walking on pathways with turns threatens stability, affecting one’s gait-related indices and posture. The ability to overcome such deficits is compromised with age and neurological disorders, e.g., Parkinson’s Disease (PD) leading to falls. Also, task demands imposed by single and dual-task (e.g., counting backward while walking) conditions affect the gait of individuals using different postural strategies varying with age and neurological disorder. Existing research has investigated either the effect of the pathway with turn or task condition on one’s gait. However, none (to our knowledge) have explored the differentiated implications of the pathway with turn and task conditions on one’s gait-related indices and knee flexion while walking. Our study had two phases with 30 participants. Phase 1 had healthy adults (young and old) and Phase 2 had age and gender-matched healthy elderly and individuals with Parkinson’s disease (PD) who walked on pathways having turns under single and dual-task conditions. We analysed gait in terms of (i) gait-related indices (Phases 1 and 2) and (ii) knee flexion (Phase 2). Also, we analysed one’s counting performance during dual task. One’s gait-related indices and knee flexion were measured using a portable gait quantifier. The aim was to (i)understand whether both pathways with turn and task conditions are equally effective in affecting the gait of (a)individuals of varying ages and (b) gender-matched healthy older adults and individuals with PD, (ii)study variations of knee joint angles while walking on pathways having turns (under different task conditions) in terms of its clinical relevance, and (iii) explore the implication of pathway with turn on counting performance (with relevance to postural strategy) with varying age and PD. Results indicated that for the younger group, the task condition caused statistical variations in gait-related indices. For the older group, both pathways with turn and task conditions had statistical implications on gait-related indices. Additionally, individuals with PD demonstrated a higher variation in knee flexion than their healthy counterparts. Again, pathways with varying turns elicited variations in counting performance indicating different postural strategies being employed by the three groups.

## Introduction

One’s gait (describing the pattern of walking) is an indicator of the quality of life (Paraschiv-Ionescu et al., 2004), with gait being influenced by age (Hodgins, 2008), pathway turn angle (de Morais Faria et al., 2016), task demands (Bayot et al., 2020), etc. Often, the elderly demonstrate slower walking (Hodgins, 2008) than the young, accompanied by reduced cadence (Novak et al., 2016) along with increased step time (Nagano et al., 2013) and variations in knee flexion (Begg and Sparrow, 2006) as mechanisms to achieve dynamic stability and prevent falls. Again, walking on pathways having different trajectories, namely straight or with turns (de Morais Faria et al., 2016) can influence one’s gait (with the influence increasing with the turning angle (de Morais Faria et al., 2016) with variations quantified in terms of changes in gait-related indices, e.g., stride time, step time, cadence, etc. (O’Sullivan et al., 2013), and knee flexion defining one’s posture during walking (Hora et al., 2017). Turning on a curve along a pathway not only threatens stability but also requires a precise balance of each limb (Bhatt et al., 2013) (affecting one’s gait-related indices and posture), and such an ability is often compromised with an increase in age (Shin et al., 2015) and neurological disorders, such as PD (Bhatt et al., 2013). This is because, turning on a curve (a complex and difficult maneuver for elderly individuals) demands changes in both anteroposterior and medo-lateral impulses, while moving one’s centre of mass toward the new direction of travel (Akram et al., 2010) and precise control of medo-lateral balance is important for preventing falls during walking (Bergland et al., 2003; Jansen et al., 2014). Also, adding to anomalies in gait-related indices (Bhatt et al., 2013), the individuals with PD often demonstrate abnormal posture (Yoshii et al., 2016) with knee flexion reported as one of the observations in their stereotypical stooped appearance (Yoshii et al., 2016). Such flexed posture might adversely affect their daily living and can be associated with falls (Yoshii et al., 2016). The impact of these falls can be debilitating not only because of associated injuries, but also the secondary immobilisation caused by a fear of renewed falls (Bloem et al., 2006).

Adding to the pathway turns, the task demands imposed by task conditions, e.g., single task and dual task conditions (Bayot et al., 2020) during walking can have implications on one’s gait. Research has shown that different task conditions, e.g., walking while reciting alternate letters of the alphabet, serial subtraction, counting backward, etc. (Bayot et al., 2020), can have varying implications on one’s gait. Such implications can be at least partially attributed to the cognitive load due to the dual task (Bayot et al., 2020) that can cause individuals to use different postural strategies varying with age and neurological disorder. For example, healthy young adults who enjoy the automaticity of walking (Clark, 2015) often prioritize cognition over walking (Plummer et al., 2015), while performing a dual task. In contrast, cognitively healthy older adults often prioritize gait over cognition (‘posture first strategy’) (Bayot et al., 2020) to maintain stability and prevent falls. Again, individuals with Parkinson’s disease tend to divide their attention between both the components of a dual task (e.g., walking and counting), while employing the ‘posture second’ strategy (Bloem et al., 2006) which might be associated with their risk of falls.

Though there is a rich history of literature in which researchers have investigated either the effect of pathway turns or task demands of healthy individuals and those with gait disorders, none (to our knowledge) have explored the differentiated implications of pathway turn angles and task conditions on gait-related indices and knee flexion during one’s overground walk with relevance to one’s postural control strategy. This gap in literature warrants deeper investigation to explore the differentiated implications of pathways with varying turn angles and task conditions on healthy and unhealthy gait. For this, we carried out a systematic study in two phases, namely phase 1 and phase 2. Phase 1 involved cognitively healthy adults belonging to younger and older age groups who participated in a walking task on pathways having varying turn angles (0, 90, 120, and 180°) under single-task and dual-task (counting backward while walking) conditions. Phase 2 involved age and gender-matched cognitively healthy elderly and individuals with PD who walked on pathways having turned and under single-task and dual-task conditions. We explored their gait in terms of (i) gait-related indices [that can characterize one’s walking (Solanki and Lahiri, 2018); in Phases 1 and 2] and (ii) knee flexion [with knee joint angle being a key component in understanding human posture (Hora et al., 2017) while walking; in Phase 2]. In addition, we analysed one’s counting performance during the dual task. One’s gait-related indices and knee flexion were measured using a portable gait quantifier (SmartWalk *henceforth*). The purpose of our study was to (i) understand whether both the pathway with varying turn angles and task conditions are equally effective in affecting the gait of (a) individuals belonging to different age groups and (b) age and gender-matched healthy older adults, and individuals with PD, (ii) study the variations of knee joint angles synchronized with gait events, while walking overground on pathways having turned and under different task conditions in terms of its clinical relevance, and (iii) explore the implication of pathway turn angles on one’s counting performance under the dual-task condition with relevance to postural control strategy from the perspective of age and neurological disorder, such as PD.

This paper is organized as follows: The section on materials and methods presents the system design, followed by the methodology used for the study. The section on results provides our findings obtained during the study. Finally, the section on discussion and limitations summarises the research findings and discusses the limitations of the current research, as well as the direction of future research.

## Materials and Methods

### Participants

Our study was conducted in phases, namely, phase 1 and phase 2. Table 1 shows the participants’ characteristics for phases 1 and 2. In phase 1, twenty healthy participants categorized into two groups, namely Grp_1_ (Y1–Y10; 20 ≤ Age ≤ 35 years) and Grp_2_ (O1–O10; Age > 50 years) were recruited. In phase 2, five age and gender-matched healthy elderly [Grp_3_ *henceforth*; (E1–E5; Age > 50 years)] and individuals with PD [Grp_4_ *henceforth;* (P1–P5; Age > 50 years)] were recruited. The individuals with PD were recruited from a nearby physiotherapy hospital, where they were undergoing treatment. All the individuals with PD were on medication. Enrolment of these participants was through a physiotherapist’s referral. The inclusion/exclusion criteria for the healthy participants were (i) age between 18 and 90 years, (ii) can understand the experimenter’s instructions, and (iii) have no neurological, musculoskeletal, or vestibular impairment. The individuals with PD were checked for their ability to perform the 10 m walk-test (O’Sullivan et al., 2013) while walking over-ground without any external support such as orthosis, canes, etc. Also, Falls Efficacy Scale (FES) scores (Thomas et al., 2010) were collected from the participants. The FES [having thresholds (Meimandi et al., 2021) as FES score < 22; between 22 and 28; and >28 considered as mild, moderate, and severe fear of fall, respectively] is a clinical tool that can be used to identify one’s confidence while an individual does basic tasks of daily living without falling. This is correlated with measures of balance and gait (Yardley and Smith, 2002) and predicts future falls. On average, the FES scores varied across groups with Grp_1_ having the lowest (who can be considered to be confident) and Grp_4_ having the highest score (Grp_1_ < Grp_2_ < Grp_3_ < Grp_4_ in terms of FES scores). All the participants belonging to Grp_2_ and Grp_3_ [except a few, i.e., O1, O2, O6, O7, E1, and E5 (Table 1) who demonstrated confident walking] demonstrated mild fear of falling with one, namely E4 (in Grp_3_) who demonstrated moderate fear of fall. In contrast, for Grp_4_, three of the participants [P2, P4, and P5 (Table 1)] demonstrated severe fear of falling, while one showed moderate fear of falling and one (P3) had a mild fear of falling. Again, clinical measures, such as the Unified Parkinson’s Disease Rating Scale motor part (UPDRS III) (Pandey et al., 2016) and H&Y stage (Pandey et al., 2016) indicate the severity of the symptom was collected for Grp_4_. The Mean (SD) of UPDRS III and H&Y stage is 28.60 (±8.29) and 2 (±0.71), respectively, which shows the mild severity of disease for Grp_4_ (Pandey et al., 2016). The study had institute ethical clearance (Approval No.: IEC/2014-15/2/UL/003).

**TABLE 1:** Participants’ characteristics.

Grp_1_ (phase 1)				Grp_2_ (phase 1)			
ID (gender)	Age (years)	BMI (kg/m^2^)	FES (score)	ID (gender)	Age (years)	BMI (kg/m^2^)	FES (score)
Y1 (M)	28	19	10	O1 (M)	57	20	10
Y2 (F)	27	23.3	10	O2 (M)	59	24.1	10
Y3 (M)	28	19	10	O3 (M)	68	19.1	14
Y4 (F)	33	24.5	10	O4 (M)	62	24.2	12
Y5 (M)	30	19.2	10	O5 (M)	60	20	13
Y6 (F)	29	24.1	10	O6 (F)	58	20	10
Y7 (M)	33	21.7	10	O7 (F)	55	19.6	10
Y8 (M)	35	22.7	10	O8 (M)	69	19.1	16
Y9 (F)	28	23.7	10	O9 (M)	65	24.1	14
Y10 (M)	31	23	10	O10 (M)	62	20	12
**Mean (*SD*)**	30.20 (±2.59)	22.02 (±1.90)	1.00 (±0.00)	**Mean (*SD*)**	61.50 (±4.64)	21.02 (±2.17)	12.10 (±2.13)

**Grp_3_ (phase 2)**				**Grp_4_ (phase 2)**			
**ID (gender)**	**Age (years)**	**UPDRS III/H&Y**	**FES (score)**	**ID (gender)**	**Age (years)**	**UPDRS III/H&Y**	**FES (score)**

E1 (F)	55	–	10	P1 (F)	52	18/1	23
E2 (M)	65	–	14	P2 (M)	65	40/3	49
E3 (M)	66	–	16	P3 (M)	67	24/2	18
E4 (M)	73	–	22	P4 (M)	71	29/2	29
E5 (M)	67	–	10	P5 (M)	65	32/2	40
**Mean (*SD*)**	65.20 (±6.49)	–	14.4 (±4.97)	**Mean (*SD*)**	64.00 (±7.14)	28.60/2 (±8.29/±0.71)	31.80 (±12.64)

*ID, participants’ ID; BMI, Body Mass Index; UPDRS III, Unified Parkinson’s Disease Rating Scale motor part; FES, Falls Efficacy Scale; M, male; F, female.*

### Experimental Setup

**FIGURE 1:**
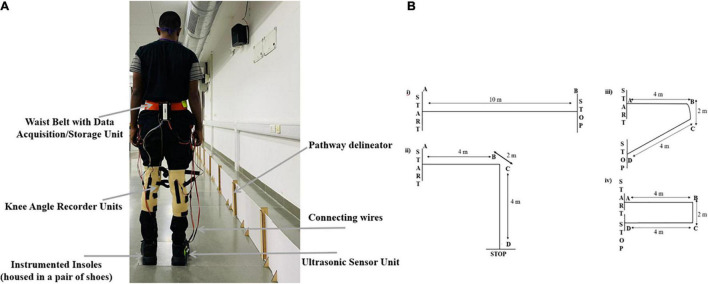
**(A)** Back view of a person wearing SmartWalk, **(B)** 10-m pathways (i) 0° (A–B), (ii) 90° (A–D), (iii) 120° (A–D), and (iv) 180° (A–D). AB and CD are straight segments and BC is turn segment. Note: Knee Angle Recorder Units were used in Phase 2 of our study.

The experimental setup for both the phases 1 and 2 comprised of (i) a SmartWalk system comprising of a waist-belt (housing the Data Acquisition/Storage Unit), Instrumented Insoles (housed in a pair of shoes), and *Knee Angle Recorder Units* positioned in a pair of knee caps that were adjustable with Velcro belts (Figure 1A) for each leg (Figure 1A; described earlier) and (ii) 10-m pathway identified by a pathway delineator (having markers every 1 m), along with ‘START’ and ‘STOP’ lines (Figure 1B). Please note that the *Knee Angle Recorder Unit* of the SmartWalk system was used only in Phase 2. The pathway was of 4 types based on the pathway turn angles, e.g., 0, 90, 120, and 180°, with each pathway having straight segments and a turn segment, except 0° (that had only the straight segment).

### Procedure

Each phase of our study had single task (Task_C1_ *henceforth*) and dual-task (Task_C2_ *henceforth*) conditions. In Task_C1_, one was asked to walk (with self-selected speed) on the pathway without speaking. In Task_C2_, one was asked to walk (with self-selected speed) on the pathway while counting backward (Bayot et al., 2020) and the counting performance was recorded by the experimenter. One was told not to match his/her steps with backward counting. The order of presentation of Task_C1_ and Task_C2_ was randomized across participants to eliminate ordering effects (Bayot et al., 2020). Each of Task_C1_ and Task_C2_ required ∼30 min of commitment from each participant. In each phase, when the participant entered the study room, the experimenter asked him/her to sit on a chair and relax. This was followed by the experimenter showing the experimental setup to the participant and demonstrating what he/she was expected to do in the study while describing the study with a visual schedule (abiding by the ethical considerations). Also, one was told that he/she was free to discontinue the study at any time if uncomfortable. In addition, they were told that they can ask for a break at any point during the tasks. When the participant expressed that he/she understood the task and consented to participate in the study, the consent signing was administered. In the case of Phase 2 for Grp_4_, the accompanying therapist collected the clinical measures. Also, the therapist confirmed whether the patient was in the OFF-state (for data collection). Then, the experimenter helped the participant to wear the SmartWalk and offered the pathways for walking with components of SmartWalk and pathways varying depending on the Phase of the study. At the end of each phase, the experimenter collected verbal feedback on the overall impression of the participant regarding the use of SmartWalk and his/her participation in the study.

#### Procedure for Phase 1

In this phase, the participant was offered pathways with 0, 90, 120, and 180° turns (order of presentation being randomized across participants). The experimenter helped the participant to wear the waist belt (housing the Data Acquisition/Storage Unit), shoes (housing the *Insoles*), and Ultrasonic Sensor Unit (mounted on the lateral side of the heel location of one of the shoes). Also, before commencing a walk in each of Task_C1_ and Task_C2_, the participant was asked to stand as straight as possible with both legs touching the ‘START’ line (Figure 1B).

#### Procedure for Phase 2

In this phase, the participant was offered the pathway having 0 and 180° turns (Figure 1B). The experimenter helped the participant to wear the waist belt, shoes, Ultrasonic Sensor Unit, and a pair of kneecaps (adjustable with Velcro belts) housing the *Knee Angle Recorder Units* toward the knee pit. Also, before commencing walk in each of Task_C1_ and Task_C2_, the participant was asked to stand as straight as possible with both legs touching the ‘START’ line (Figure 1B) when baseline recording of the Knee flexion (Angle_Baseline_ *henceforth*) was made.

### SmartWalk – Its Design

The SmartWalk (Figure 1A) comprised of (A) Instrumented Insoles, (B) Ultrasonic Sensor Unit, (C) Flexion/Extension Recorder Unit (*Knee Angle Recorder Unit* henceforth), and (D) Data Acquisition/Storage Unit.

In our present research, we used a pair of Instrumented Insoles (*Insoles* henceforth) that were housed in a pair of shoes. The idea was to use a wearable and portable gait quantifier since the existing state-of-the-art stereophotogrammetric systems and walk mats are used to quantify one’s gait-related indices computed from gait events, namely, heel-strike, toe-off, etc. (O’Sullivan et al., 2013), though powerful, suffer from large setup time, operational complexity, specialized knowledge for operation, high cost, restriction to lab-based settings, line-of-sight issues, etc. Again, within the wearable and portable devices are accelerometers, gyro sensors, goniometers, etc. (Lan and Shih, 2014) that though powerful, often suffer from drift problems (Lan and Shih, 2014) that limit their application in gait monitoring. To overcome such issues, researchers have been exploring the use of portable force sensors (Hanlon and Anderson, 2009) such as Force Sensitive Resistors (FSRs) to detect one’s gait events and measure gait-related indices. For example, different researchers have used multiple FSRs located at different positions under one’s feet (Pinkam and Nilkhamhang, 2013) to characterize one’s gait. Usage of multiple FSRs though lends improved precision of measurements, yet it increases the hardware complexity along with difficulty in troubleshooting making it infeasible for practical applications. On the other hand, too few FSRs can miss picking up certain aspects of gait abnormalities, such as foot inversion/eversion (Forghany et al., 2014) that can be seen in the elderly and those with gait disorders. In our present research, each of the *Insoles* had FSRs [0–445°N (FlexiForce A201; from Tekscan) with an active diameter of 9.53 mm] [similar to that mentioned in Solanki and Lahiri (2018)] and the data was acquired from sensors placed at the heel locations [lateral and medial border to take care of any possible foot inversion/eversion (Forghany et al., 2014)] to record one’s heel-strike events. The acquired data was processed to compute one’s gait-related indices, namely, Stride time (O’Sullivan et al., 2013), Step time (O’Sullivan et al., 2013), and cadence (O’Sullivan et al., 2013) (details in Section “Data Processing” below). The *Insoles* were calibrated using the VICON setup (from Vicon Motion Systems Ltd.). The calibration results of a pilot study with healthy participants {*n* = 5; Mean (SD) = 27 (±3.67) years; Body Mass Index (BMI) [Mean (*SD*) = 21.98 (±2.33) Kg/m^2^]} indicated good agreement between gait-related indices measured by the *Insoles* and the VICON setup with average %Absolute error being 0.71 and 0.8% for Stride time and Step time, respectively.

An Ultrasonic Sensor Unit [HCSR04 (Hanan et al., 2019)] is comprised of an ultrasonic transmitter, receiver, and control circuit. This unit was used to transmit synchronising markers to the Data Acquisition/Storage Unit to keep track of the distance traversed (by a participant) along the pathway laid out using a pathway delineator (please see Section “Experimental Setup” for details).

Again, given that the estimation of joint angle is a key component of the analysis of human gait (Lee et al., 2016), we used a calibrated *Knee Angle Recorder Unit* [similar to that mentioned in Pallavi et al. (2021)]. One’s joint angles can be measured by using standard camera-based techniques, e.g., VICON (McGrath and Stirling, 2020) which though powerful, suffers from portability issues, high cost, line-of-sight issues, etc. Given the importance of joint angle estimation and the need to have portable, wearable sensing to facilitate human gait analysis in free-living conditions, research had been focused on using Inertial Measurement Units (IMUs), Goniometers, etc. However, the IMU-based systems suffer from drift problems (Lee et al., 2016) and Electro-Goniometers may not be suitable for real-time measurement (Lee et al., 2016). To mitigate such issues, our *Knee Angle Recorder Unit* was made using a commercially available 4.5” bend sensor (from Spectra Symbol) (Figure 1A) to record the flexion/extension angle at the knee of each leg. The idea was to measure the angle between the line connecting the greater trochanter and knee, and the line connecting the knee and lateral malleolus (Yoshii et al., 2016). The bend sensor was calibrated using a stepper motor-hinge setup with a motor driver for varying bend positions (Pallavi et al., 2021). Given that the knee flexion during one’s gait is less than 90° (Rowe et al., 2000), we mounted the bend sensor in a kneecap, so that the bending location allowed us a range of measurement from 0° to ∼100° with the calibrated sensor output changing linearly (*R*^2^ = ∼0.99) with bend angle along with satisfactory repeatability (Pallavi et al., 2021). The analogue signal (0–5 V) from the bend sensor along with time stamping was acquired by the Data Acquisition/Storage Unit.

Finally, a Data Acquisition/Storage Unit housing (i) a Microcontroller (ATMEGA 2560) and (ii) a 64-GB SD card (from SanDisk Ultra), mounted on a waist-belt was used to store the analogue signals (0–5 V) from the FSRs (of the *Insoles*), Ultrasonic Sensor, and *Knee Angle Recorder Unit*. The data from the sensors were sampled using a 10-bit Analogue-to-Digital converter of the Microcontroller at ∼200 samples/second (Solanki and Lahiri, 2018) and then stored in the SD card along with time stamping. In addition, for the *Insole* data, the *Insole* ID (‘left’ and ‘right’) was stored. The stored data was further processed.

### Data Processing

The stored data were processed using the Microcontroller of the Data Acquisition/Storage Unit to extract the (i) gait-related indices and (ii) Coefficient of Variation (%CV *henceforth*) of the knee flexion during heel-strike.

### Extraction of Gait-Related Indices

The FSR data acquired from the *Insoles* was processed by the Microcontroller [as in Solanki and Lahiri (2018)] of the SmartWalk system to compute (a) Normalised Average Stride time (Stride time_AVG_NORM_ *henceforth*), Normalised Average Ste time (*Steptime*_AVG_NORM_*henceforth*) and Normalised Cadence (Cadence_NORM_ *henceforth*).

The Stride time was computed from the time interval between two consecutive heel-strike events of each ipsilateral i.e., same leg (O’Sullivan et al., 2013) followed by normalization using Equation (1) (Angelini et al., 2016). Here, *h* and *g* represent one’s body height and gravitational acceleration, respectively.


(1) $$\mathrm{Stride}\,{\mathrm{time}}_{\mathrm{AVG}\,\_\,\mathrm{NORM}}=\frac{\mathrm{Stride}\,{\mathrm{time}}_{\text{AVG}}}{\sqrt{\left(\frac{\text{h}}{\text{g}}\right)}}$$

Again, the Step time was computed from the time interval between two consecutive heel-strike events of contralateral legs (O’Sullivan et al., 2013) followed by normalization using Equation (2). The *h* and *g* are the same as before.


(2) $$\mathrm{Step}\,{\mathrm{time}}_{\mathrm{AVG}\,\_\,\mathrm{NORM}}=\frac{\mathrm{Step}\,{\mathrm{time}}_{\text{AVG}}}{\sqrt{\left(\frac{\text{h}}{\text{g}}\right)}}$$

Also, one’s cadence was computed from the heel-strike events quantifying the number of steps taken per minute (O’Sullivan et al., 2013) followed by normalization using Equation (3) (Fang et al., 2018).


(3) CadenceNORMi=Cadencei*(hi1/n⁢∑j=1nhj)

Here, *h* and *n* represent one’s body height and the number of participants, respectively.

### Extraction of Coefficient of Variation (%CV) of Knee Flexion

The coefficient of variation (of the knee flexion) can be a valuable measure with regard to falls (Moon et al., 2015) during walking. We computed the %CV of the knee flexion during heel-strike events using Equation (4).


(4) $$\%\mathrm{CV}=\frac{\mathrm{Standard}\,\mathrm{deviation}}{\text{Mean}}*100$$

### Statistical Analysis

Post our studies, we analysed the gait-related indices (computed for each of Task_C1_ and Task_C2_ during phases 1 and 2) and the counting performance (computed for Task_C2_ during Phases 1 and 2) corresponding to pathways with varying turns for the participant groups for statistical significance. Since our sample size was limited and data was not normally distributed [using the Shapiro–Wilk test (Field, 2013)], we implemented a non-parametric dependent sample Wilcoxon Signed Rank test (Field, 2013) and independent sample Mann Whitney test (Field, 2013) for investigating the statistical significance during data analysis. The statistical tests were carried out using the SPSS Statistics 20 software and effect size (*r*) was computed from the *z*-value obtained using the statistical tests (Fritz et al., 2012).

## Results

While the participants took part in our study, at the end of each phase, the experimenter collected verbal feedback from the participant that indicated that all the participants (including those belonging to Grp_4_) were comfortable with our SmartWalk. Regarding the participation in the study, Grp_1_ reported that they were comfortable with taking part in our study while walking on the pathways with varying turns and under different task conditions. For Grp_2_ and Grp_3_, we received similar feedback. For Grp_4_, the participants mentioned that they had a good experience while walking on the pathways under varying task conditions. None of them took any intermediate break during the task though they were free to ask for breaks if needed during the study. The aim of our study was to understand the implications of pathways with turns and task conditions on one’s (i) gait-related indices (measured using SmartWalk), (ii) postural changes, and (iii) dual-task performance (i.e., counting performance) with relevance to postural strategy.

### Gait-Related Indices of Grp_1_ and Grp_2_ for Pathways With Varying Turn and Task Conditions in Phase 1

Here we present our observations on the gait-related indices of Grp_1_ and Grp_2_ for pathways with varying turns and task conditions. The Grp_1_ and Grp_2_ differed statistically (*p*-value < 0.00001) with a large effect (*r* = 0.8) in age (using independent sample Mann–Whitney test).

### Gait-Related Indices While Traversing Pathways With Varying Turns: For Task_C1_

**FIGURE 2:**
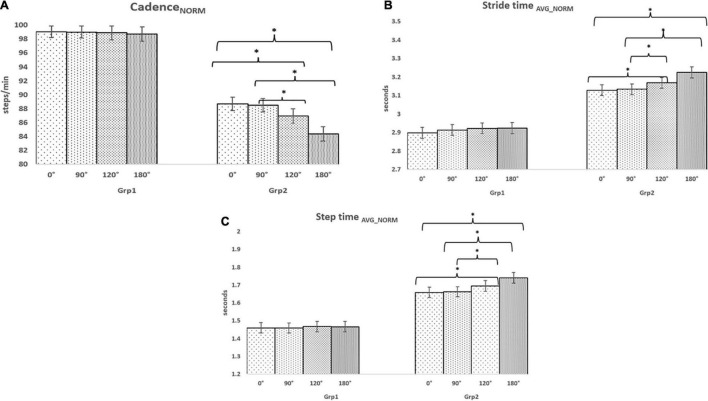
Comparative analysis of Group (Grp_1_ and Grp_2_) average **(A)** Cadence_NORM_ for Task_C1_, **(B)** Stride time_AVG_NORM_ for Task_C1_, and **(C)** Step time_AVG_NORM_ for Task_C1_. *p < 0.05.

The intra-group analysis for Task_C1_ indicated that the group average Cadence_NORM_ (Figure 2A) decreased as the turn angle of pathways increased and Stride time_AVG_NORM_ (Figure 2B) and Step time_AVG_NORM_ (Figure 2C) increased as the turn angle of pathways increased (less variation given that the participants were healthy) for each of Grp_1_ and Grp_2_. The dependent sample Wilcoxon Signed Rank test indicated that the pathways with varying turns did not cause any statistical variation in any of the gait-related indices for Grp_1_. In contrast, for Grp_2_, we could find the statistical difference (with a *p*-value ranging from 0.01 to 0.04) with a large effect (ranging from *r* = 0.52 to *r* = 0.8) in the gait-related indices, while walking on pathways having 120 and 180° turns concerning that with 0 and 90° turns.

While considering inter-group analysis, we could find that irrespective of the pathways with varying turns, there was a reduction (%Δ = 10.45%, %Δ = 10.58%, %Δ = 12.04%, and %Δ = 14.53% for 0, 90, 120, and 180°, respectively) in group average Cadence_NORM_ from Grp_1_ to Grp_2_ and an increase in Stride time_AVG_NORM_ (%Δ = 7.54%, %Δ = 7.98%, %Δ = 8.39%, and %Δ = 10.28% for 0, 90, 120, and 180°, respectively) and Step time_AVG_NORM_ (%Δ = 13.60%, %Δ = 13.98%, %Δ = 15.52%, and %Δ = 18.65% for 0, 90, 120, and 180°, respectively) from Grp_1_ to Grp_2_. The Independent sample Mann–Whitney test indicated that all the gait-related indices were statistically different (with *p*-value ranging from 0.01 to 0.03) with a large effect (ranging from *r* = 0.7 to *r* = 0.8) between Grp_1_ and Grp_2_ irrespective of pathways with varying turn angles.

### Gait-Related Indices While Traversing Pathways With Varying Turns: For Task_C2_

**FIGURE 3:**
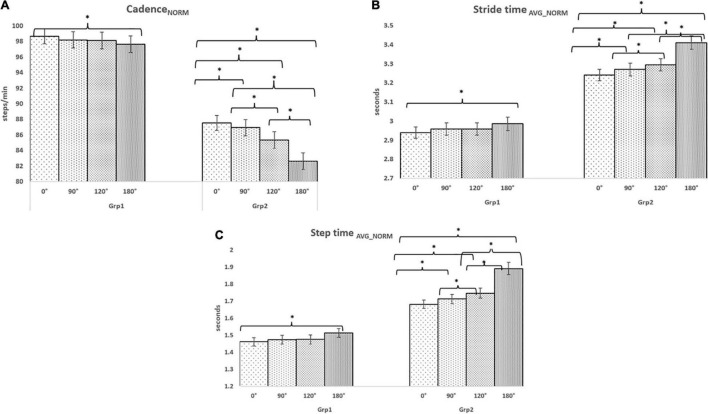
Comparative analysis of Group (Grp_1_ and Grp_2_) average **(A)** Cadence_NORM_ for Task_C2_, **(B)** Stride time_AVG_NORM_ for Task_C2_, and **(C)** Step time_AVG_NORM_ for Task_C2_. *p < 0.05.

The intra-group analysis for Task_C2_ indicated that the group average Cadence_NORM_ (Figure 3A) decreased as the turn angle of the pathways increased and the Normalised Stride time (Figure 3B) and Step time (Figure 3C) increased as the turn angle of the pathways increased for each of Grp_1_ and Grp_2_ (similar to Task_C1_). Again, from Figures 2A, 3A, we find that for pathways with varying turns, the Cadence_NORM_ for Task_C1_ was higher (%Δ = 0.40, 0.83, 0.80, and 1.08% for 0, 90, 120, and 180°, respectively, for Grp_1_ and (%Δ = 1.31, 1.82, 1.92, and 2.09% for 0, 90, 120, and 180°, respectively, for Grp_2_) than that for Task_C2_. Similar was the observation in terms of increase in the Normalised Stride and Step times from Task_C1_ to Task_C2_. A dependent sample Wilcoxon Signed Rank test revealed statistical variation (with a *p*-value ranging from 0.03 to 0.04) with a large effect (ranging from *r* = 0.66 to *r* = 0.69) in all the gait-related indices between 0 and 180° for Grp_1_ during Task_C2_, unlike that in Task_C1_. In contrast, for Grp_2_, all the gait-related indices corresponding to pathways with varying pathway turn angles were statistically different (with *p*-value ranging from 0.01 to 0.04) with a large effect (ranging from *r* = 0.7 to *r* = 0.9).

While considering the inter-group analysis, we could find that irrespective of pathway turn, there was a reduction in group average Cadence_NORM_ (%Δ = 11.90 and 12.77%, respectively, for Task_C1_ and Task_C2_) from Grp_1_ to Grp_2_ and an increase in Stride time_AVG_NORM_ (%Δ = 8.54 and 11.58%, respectively, for Task_C1_ and Task_C2_) and Step time_AVG_NORM_ (%Δ = 15.43 and 18.64%, respectively, for Task_C1_ and Task_C2_) from Grp_1_ to Grp_2_. The Independent sample Mann–Whitney test indicated that all the gait-related indices were statistically different (with *p*-value ranging from 0.01 to 0.04) with a large effect (ranging from *r* = 0.52 to *r* = 0.9) between Grp_1_ and Grp_2_ corresponding to pathways with varying turn angles. Again, while comparing the observations for Task_C1_ and Task_C2_, the %Δ in the gait-related indices between Grp_1_ and Grp_2_ during Task_C2_ was higher than that for Task_C1_.

### Comparative Presentation of Gait-Related Indices of Grp_1_ and Grp_2_ for Pathway With Varying Turns and Task Conditions in Phase 1

We wanted to understand whether both the pathways having varying turns and task conditions were equally effective in affecting the gait of Grp_1_ and Grp_2_. Irrespective of the task condition, pathways having varying turns contributed to statistically significant variation (with *p*-value ranging from 0.01 to 0.03) with a large effect (ranging from *r* = 0.7 to *r* = 0.8) using Dependent sample Wilcoxon Signed Rank test) in the gait-related indices for Grp_2_ unlike that for Grp_1_ (with no statistical difference in the indices except that between pathways with 0 and 180° turns for Task_C2_).

Again, irrespective of the pathway having varying turns, variation in the task condition contributed to statistically significant variation (with a *p*-value ranging from 0.01 to 0.048) with a large effect (ranging from *r* = 0.52 to *r* = 0.9) using dependent sample Wilcoxon Signed Rank test in the gait-related indices for Grp_2_ and Grp_1_. In summary, both the pathways (with turns) and task condition were effective in affecting the gait of the older group (Grp_2_) unlike that of the younger group (Grp_1_), whose gait was more affected by the variation in task condition than that due to pathway having varying turn angles. However, given the small sample size, we do not intend to generalize our observations.

### Gait-Related Indices of Grp_3_ and Grp_4_ for Pathways With Varying Turn and Task Conditions in Phase 2

Having seen that the increase in pathway turn contributes to variation in one’s gait-related indices and that the 180° pathway turn caused statistical variations in the gait-related indices of even the healthy young adults, concerning that with 0° turn in the case of Task_C2_, we wanted to understand the implication of pathways with 0 and 180° turn angles on the gait-related indices of age and gender-matched healthy elderly (Grp_3_) and individuals with PD (Grp_4_) through a pilot study (i.e., phase 2). Also, we wanted to carry out a comparative investigation of the effect of task conditions on their gait-related indices. Again, given that knee flexion is associated with a stereotypical stooped appearance in individuals with PD (Yoshii et al., 2016) that might be related to falls (Yoshii et al., 2016), we wanted to understand the implication of pathways with varying turn angles and task condition on the knee flexion measured by the *Knee Angle Recorder Unit*.

### Gait-Related Indices While Traversing Pathways With Varying Turns: For Task_C1_

**FIGURE 4:**
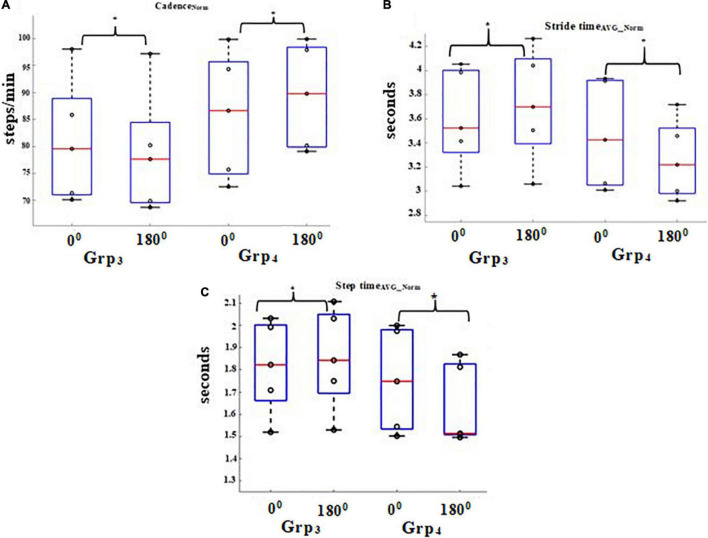
Comparative analysis of Group (Grp_3_ and Grp_4_) average **(A)** Cadence_NORM_ for Task_C1_, **(B)** Stride time_AVG_NORM_ for Task_C1,_ and **(C)** Step time_AVG_NORM_ for Task_C1_. *p < 0.05.

The intra-group analysis for Task_C1_ indicated a reduction (%Δ = 2.78%) in the group average Cadence_NORM_ (Figure 4A) with an increase in turn angle of pathways for Grp_3_ (similar findings as discussed earlier for Grp_2_) and an increase in Stride time_AVG_NORM_ (%Δ = 3.06%) (Figure 4B) and Step time_AVG_NORM_ (%Δ = 2.04%) (Figure 4C) with an increase in turn angle of pathways for Grp_3_ (similar findings as discussed earlier for Grp_2_). In contrast, for Grp_4_, an increment (%Δ = 4.14%) in the group average Cadence_NORM_ (Figure 4A) for pathway with increasing turn angles and a decrease in Stride time_AVG_NORM_ (%Δ = 5.93%) (Figure 4B) and Step time_AVG_NORM_ (%Δ = 6.46%) (Figure 4C) for pathway with increasing turn angles were observed. A dependent sample Wilcoxon Signed Rank test revealed statistical variation (*p*-value = 0.04) with a large effect (*r* = 0.9) in all the gait-related indices while considering pathways with 0 and 180° turns for both groups during Task_C1_. In addition, we wanted to understand the clinical relevance of such statistical variations in the gait-related indices of Grp_4_ in terms of its relation to FES scores. Pearson’s correlation (Ratner, 2009) between their FES scores and gait-related indices on an average was ∼0.70 for pathway with 0^0^ turns and ∼0.64 for that with 180° turns.

### Gait-Related Indices While Traversing Pathways With Varying Turns: For Task_C2_

**FIGURE 5:**
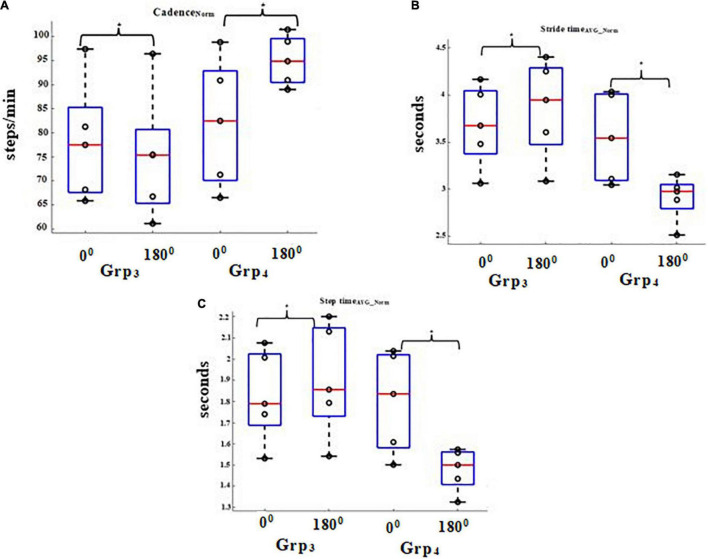
Comparative analysis of Group (Grp_3_ and Grp_4_) average **(A)** Cadence_NORM_ for Task_C2_, **(B)** Stride time_AVG_NORM_ for Task_C2,_ and **(C)** Step time_AVG_NORM_ for Task_C2_. *p < 0.05.

The intra-group analysis for Task_C2_ indicated a reduction (%Δ = 3.85%) in the group average Cadence_NORM_ (Figure 5A) with an increase in the turn angle of pathways for Grp_3_ (similar to that in Task_C1_ above) and an increase in the Normalised Stride time (%Δ = 4.89%) (Figure 5B) and Step time (%Δ = 4.12%) (Figure 5C) for pathways with increasing turn angle for Grp_3_ (similar to that in Task_C1_ above). In contrast, for Grp_4_, we can see that there was an increment (%Δ = 15.92%) in the group average Cadence_NORM_ (Figure 5A) with an increase in the turn angle of pathways (similar to that in Task_C1_) and a decrease in Stride time_AVG_NORM_ (%Δ = 17.94%) (Figure 5B) and Step time_AVG_NORM_ (%Δ = 17.83%) (Figure 5C) with an increase in turn angle of pathways (similar to that in Task_C1_). However, the variation in the group average values of all the gait-related indices for pathways with 0 and 180° turns was much higher in Task_C2_ when compared with Task_C1_, showing the increased effect of Task_C2_ on one’s fall risk than the Task_C1_. Given that the number of participants in each group is small, we do not want to generalize our findings. A dependent sample Wilcoxon Signed Rank test revealed statistical variation (*p*-value = 0.04) with a large effect (*r* = 0.9) in all the gait-related indices while considering pathways with 0 and 180° turns for both groups during Task_C2_ (similar to that in Task_C1_). About the clinical relevance of such statistical variations in the gait-related indices of Grp_4_ in terms of its relation to FES scores, we found that on an average the correlation was ∼0.74 for pathway with 0° turns and ∼0.61 for that with 180° turns.

### Variation of Knee Flexion for Grp_3_ and Grp_4_

In gait analysis, variability is commonly understood in terms of the fluctuation in the value of a joint angle (Chau et al., 2005). We were interested in further investigating the variability in the knee flexion measured by the *Knee Angle Recorder Unit* of Grp_3_ and Grp_4_ during the heel-strike event while walking on pathways having 0 and 180° turns under both the task conditions since the heel-strike is an important contributor to the bipedal stability (O’Sullivan et al., 2013). Here, we present the group average Coefficient of Variation (%CV; Section Extraction of Gait-related Indices) of the knee flexion of participants during the heel-strike event.

**FIGURE 6:**
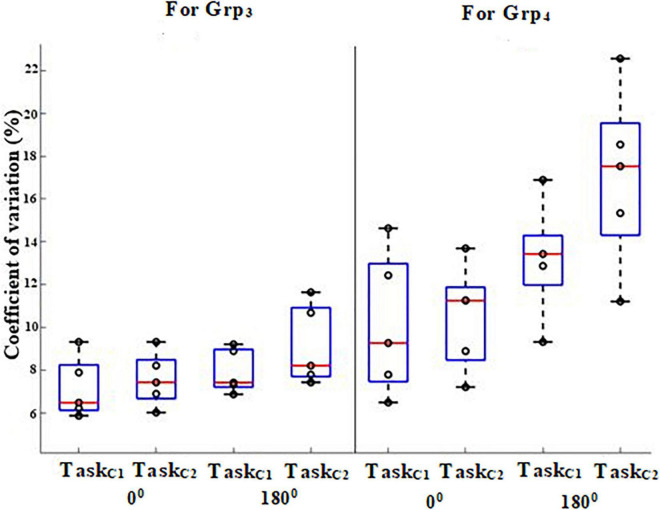
%CV of knee flexion angle while walking on 0 and 180° during heel-strike of Grp_3_ and Grp_4_.

It can be seen from Figure 6 that the %CV of the knee flexion for Grp_4_ was found to be statistically higher (*p*-value = 0.04) with a large effect (*r* = 0.9) for pathway with 180° turns than that with 0° turns for each of Task_C1_ and Task_C2_ (i.e., %Δ = 41.48 and 66.05% for 0 and 180°, respectively, for Task_C1_ and %Δ = 38.13 and 86.18% for 0 and 180°, respectively, for Task_C2_) when compared with age and gender-matched healthy elderly irrespective of task conditions. Additionally, the pathway with 180° turn led to increased variation in the knee flexion particularly for Grp_4_, and these variations were found to be higher for Task_C2_ than that for Task_C1_ (i.e., %Δ = 30.29 and 62.83% for Task_C1_ and Task_C2_). About the clinical relevance of such variations in the knee flexion of Grp_4_ in terms of its relation to FES scores, we found that on average, the correlation was ∼0.68 for pathway with 0° turns and ∼0.62 for that with 180° turns. However, the correlation between the %CV of the knee flexion and FES scores does not necessarily imply that the %CV of the knee flexion (that is considered as a possible proxy measure of bipedal instability of an individual) is a cause behind the bipedal instability. Given that the number of participants in Grp_4_ was small, we do not want to generalize our findings.

### Knee Flexion (Reflecting Postural Change) for Different Pathway Segments

After understanding the implication of variation in knee flexion during heel-strike events on one’s gait, we wanted to do a deeper investigation of the contribution of the pathway segments [e.g., straight and turn segments (Figure 1B)] on the knee flexion of Grp_3_ and Grp_4_, while walking overground on pathways having 0 and 180° turns for both the task conditions. For this, we computed the average %change in knee flexion from the respective Angle_Baseline_ measure (see Section “Procedure”) of Grp_3_ and Grp_4_ while utilizing the synchronising markers issued by the Ultrasonic Sensor Unit (Figure 1A) tracking the distance (*d* say) traversed (by a participant) along the pathway laid out using the pathway delineator for (i) 0 m ≤ *d* ≤ 1 m, (ii) 1 m < *d* ≤ 4 m, (iii) 4 m < *d* ≤ 6 m, and (iv) 6 m < *d* ≤ 10 m.

**FIGURE 7:**
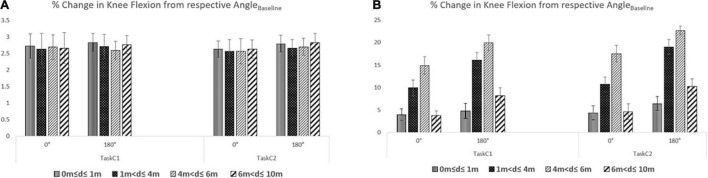
%Change of knee flexion from respective Angle_Baseline_ while walking overground on 0 and 180° **(A)** for Grp_3_ and **(B)** for Grp_4_.

It can be seen from Figures 7A,B that the %change in knee flexion from the respective Angle_Baseline_ measure (irrespective of the task condition and pathways with 0 and 180° turns) was higher for Grp_4_ than that of Grp_3_ corresponding to each pathway segment. Again, for Grp_4_, while considering the pathway with 180° turn, the %change in knee flexion from the respective Angle_Baseline_ was maximum for the turn segment (i.e., 4 m < *d* ≤ 6 m) for each of Task_C1_ and Task_C2_ (Figure 7B). However, the %change in knee flexion from respective Angle_Baseline_ was found to be higher in Task_C2_ (irrespective of the pathway segment) than that in Task_C1_ (average %Δ = 13.45 and 22.60%, respectively, for pathways with 0 and 180° turns). In contrast, for Grp_3_ (Figure 7A), in Task_C2_, we saw relatively less %change in knee flexion from the respective Angle_Baseline_ (irrespective of the pathway segments) than that in Task_C1_ (average %Δ = −2.83 and −2.39%, respectively, for pathways with 0 and 180° turns). Given that the number of participants in both groups was small, we do not want to generalize our findings.

### Counting Performance While Traversing Pathways With Varying Turns: Relevance to Postural Control Strategy

**FIGURE 8:**
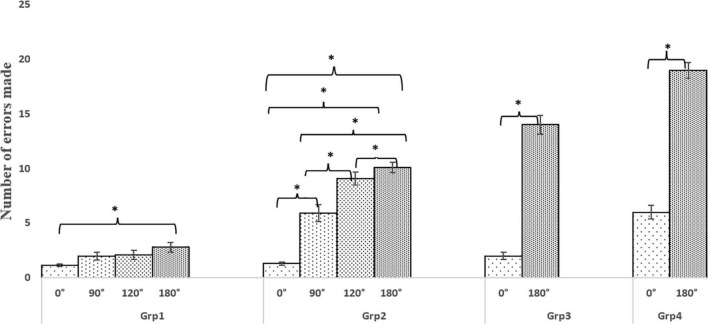
Comparative group analysis of implications of task conditions on the errors made by Grp_1_, Grp_2_, Grp_3_, and Grp_4_. *p-value < 0.05.

During Task_C2_, the participants counted backward while walking on pathways having varying turn angles. Their counting performance (in terms of the number of errors made in counting) was analysed and is shown in Figure 8. While in phase 1 of the study, Grp_1_ and Grp_2_ walked on pathways having 0, 90, 120, and 180° turns, in phase 2, Grp_3_ and Grp_4_ walked on the pathways having 0 and 180° turns. We can see that Grp_4_ made the maximum number of counting errors corresponding to the pathways traversed by them with Grp_3_, making more counting errors than Grp_2_, who, in turn, made more errors than Grp_1_ with an inter-group statistical difference (with *p*-value ranging from 0.040 to 0.048) with large effect (ranging from *r* = 0.65 to *r* = 0.69) based on an independent sample Mann–Whitney test (except for pathway with 0° turn between Grp_1_ and Grp_2_, between Grp_1_ and Grp_3_, and between Grp_2_ and Grp_3_).

Again, considering the intra-group analysis (with dependent sample Wilcoxon Signed Rank test), for Grp_1_, we found that the counting performance was statistically different (*p*-value = 0.048) and large effect (*r* = 0.8) only between pathways with 0 and 180° turns. For Grp_2_, the counting performance was statistically different (*p*-value = 0.048) with a large effect (*r* = 0.7) across all the pathways with 0, 90, 120, and 180° turns. Finally, for each of Grp_3_ and Grp_4_, the difference in the counting performance between pathways with 0 and 180° turns was statistical (*p*-value = 0.048) with a large effect (*r* = 0.8).

In summary, a higher number of counting errors made by the elderly groups (Grp_2_, Grp_3_, and Grp_4_) can be an indicator of reduced cognitive performance (Maclean et al., 2017) under dual-task conditions (Task_C2_), particularly for the pathway with 180° turn which was accompanied with a deterioration in their gait performance (Figures 4–7). Again, the decrease in the cognitive and gait performance was the most prominent for individuals with PD (i.e., Grp_4_) among all the participants, specifically for the pathway with 180° turn under dual-task conditions. Given that the number of participants in Grp_4_ was small, we do not want to generalize our findings.

## Discussion

The main contribution of this study was to examine the implication of pathways with varying turn angles and task conditions on the gait of healthy young (Grp_1_), healthy elderly (Grp_2_ and Grp_3_) and individuals with PD (Grp_4_) by using a wearable system, namely SmartWalk that can quantify gait in terms of (gait-related indices using Instrumented *Insoles* and knee flexion using *Knee Angle Recorder Unit*). Even though there are different factors that can contribute to one’s risk of falls, our wearable system can be used to measure at least a few of these factors, namely variation in one’s gait-related indices and postural changes (in terms of knee flexion) while walking that can have relevance to one’s falls. Again, unlike previous studies that explored the implication of either walking on pathways having various turns (de Morais Faria et al., 2016) or walking under various task conditions (Bayot et al., 2020), here we have investigated the differentiated implication of walking on pathways with varying turn angles (0, 90, 120, and 180°) and task conditions [single (Task_C1_) and dual-task (Task_C2_) conditions] on one’s gait-related indices and knee flexion by carrying out a systematic study involving cognitively healthy adults belonging to younger and older age groups. In addition, we have investigated the implication of walking on pathways with turns on counting performance along with its dependence on age and also among those with PD.

In an endeavour to investigate the differentiated implication of walking on pathways with varying turn angles and task conditions on one’s gait-related indices, we first studied the effect of pathways with varying turn angles while keeping the task condition the same. For each of Task_C1_ and Task_C2_, we observed a reducing trend in the normalised cadence along with an increasing trend in normalised stride and step times due to walking on pathways with increasing turning angles for each of Grp_1_, Grp_2_, and Grp_3_. A reduction in cadence might infer an increased tendency of the healthy adults to stabilize themselves to prevent falls triggered by pathways having turns (Cao et al., 1997), with the implication being more prominent for pathways having 120 and 180° turns. The turn segments are 120 and 180°, comprising of one 90° turn [often considered to be a rapid change in direction of walk that might trigger unstable walk (Dotov et al., 2016)] along with an additional 30° turn and two 90° turns, respectively, might trigger unstable walk, thereby making these pathways leading to increasing the risk of fall (de Morais Faria et al., 2016), particularly true for Grp_2_ and Grp_3_. In-depth data analysis on stride and step times of our participants under different task conditions and pathways with turns show that for Grp_1_, the variability in the group average normalised stride time was nearly double the group average normalised step time, particularly for pathway with turning angle of 180° irrespective of the task condition. Such observation can be possibly attributed to the spin turn (Gavriliuc et al., 2019) being demonstrated by the majority (∼70%) of Grp_1_, while traversing the turn segment [i.e., 4 m < *d* ≤ 6 m; Figure 1B] of the pathway in which one altered direction by spinning his/her body about the pivot foot (Gavriliuc et al., 2019) [with the rest (∼30%) of the Grp_1_ showing step turn (Gavriliuc et al., 2019)], thereby affecting the stride made by the individual (specifically at the turn segment of the pathway). However, for Grp_2_, Grp_3_, and Grp_4_, the variability in the group average normalised stride time was nearly similar to that of step time, which can be possibly attributed to the majority of our elderly healthy participants, i.e., Grp_2_ and Grp_3_ (∼63% showing step turn; rest showing spin turn), and individuals with PD, i.e., Grp_4_ (∼100%), showed step turn [marked by a weight shift from one leg to the other, while changing the direction of walking (Gavriliuc et al., 2019)], while traversing the turn segment of the pathway to ensure greater stability (Taylor et al., 2005) and prevent possible fall. While considering all the gait-related indices specifically for Grp_4_, we saw a decrease in normalised stride and step times along with an increase in the normalised cadence due to walking on pathways with increasing turning angles. Such observation for individuals with PD can be indicative of the risk of falls (Hoskovcová et al., 2015) due to pathways with turns, which not only threatens their dynamic stability (Bhatt et al., 2013), but also can be a major contributor to triggering of the freezing (Bhatt et al., 2013) that, in turn, might lead to falls (Bhatt et al., 2013). Again, while exploring the effect of task conditions while keeping the pathways with varying turn angles the same, the variation in the group average values of all the gait-related indices was much higher in Task_C2_ than that in Task_C1_, attributed to greater cognitive load (Bayot et al., 2020) in Task_C2_ as compared to Task_C1_.

Again, to investigate the differentiated implication of walking on pathways with varying turn angles and task conditions on one’s postural changes (in terms of knee flexion), we investigated the variation in one’s knee flexion for Grp_3_ and Grp_4_. While exploring the effect of pathways having varying turn angles (0 and 180°) while keeping the task condition the same, we found that the pathway with 180° turn elicited greater variability in the knee flexion at heel-strike than that for 0° for each of Grp_3_ and Grp_4_ with the variability being higher for Grp_4_ than that of Grp_3_. Similar observations about Grp_3_ and Grp_4_ while exploring the effect of task conditions while keeping the pathways the same (i.e., pathways having either 0 or 180° turn). Such an observation might infer that Grp_4_ experienced greater turning difficulties (Bhatt et al., 2013) and also the dual-task condition (contributing to the cognitive load) might have led to increased postural instability in them (Bloem et al., 2006) compared to the age and gender-matched healthy elderly. Our findings are also in line with literature reporting that individuals with PD show more postural instabilities (Yoshii et al., 2016) than their healthy counterparts, and this might be due to greater difficulty in coordination and timing faced by them (Bloem et al., 2006) making them more prone to falls.

While we have been investigating the implications of walking on pathways having varying turn angles on one’s postural changes (in terms of knee flexion), we have been looking at the contribution of the pathway in its entirety rather than exploring the contribution of individual segments (e.g., straight and turn segments) of the pathway on one’s postural changes in Grp_3_ and Grp_4_. Our results showed that the %change in knee flexion (irrespective of the pathway segments and the task condition) was higher for Grp_4_ than that of Grp_3_ for both pathways with 0 and 180° turns. Such observations are in line with the literature (Yoshii et al., 2016) indicating that a flexed posture is often demonstrated by individuals with PD while walking. Again, the effect of the turn segment (i.e., 4 m < *d* ≤ 6 m) on the knee flexion was higher than that of the other pathway segments and this was true for each of Task_C1_ and Task_C2_ with the effect being more prominent for Task_C2_ than that for Task_C1_. This might be due to the dual task (Task_C2_), causing the individuals with PD to divide their attention between both the components (i.e., walking and counting), while employing posture second strategy (Bloem et al., 2006) associated with the risk of falls.

Added to exploring the implication of pathways with varying turn angles and task conditions on one’s gait and posture, we also explored the counting performance of all our participants. Our results show that the elderly (Grp_2_, Grp_3_, and Grp_4_) made more counting errors than that the younger group (Grp_1_) corresponding to pathways having varying turn angles with Grp_4_ making the maximum number of counting errors for pathways with 180° turns. Based on the gait-related indices [i.e., reduction in normalised cadence and increase in normalised stride and step times (Figure 3) and the counting performance (Figure 8) of Grp_2_ and Grp_3_], we can say that both of these groups prioritized gait over cognition while employing ‘posture first strategy’ (Bayot et al., 2020) to ensure stability (Novak et al., 2016), unlike Grp_1_ who enjoyed automaticity of walk (Clark, 2015) and prioritized cognition over gait (Plummer et al., 2015). In contrast, for Grp_4_, an increase in the number of counting errors irrespective of the pathways having varying turns (Figure 8) coupled with an increment in normalised cadence, along with a reduction in the normalised stride and step times (Figures 4, 5), along with increased variation in the knee flexion (Figures 6, 7) compared to Grp_3_ might infer that Grp_4_ used a “posture second” strategy and treating all elements of a complex task with equal priority, while compromising with balance, leading to falls (Yoshii et al., 2016).

In summary, in our present study, we investigated the implications of pathways with varying turns and task conditions on one’s gait-related indices, knee flexion, and counting performance in healthy young, healthy old, and age and gender-matched individuals with PD. Our results indicated that for the older group, both the pathway with varying turns and the task conditions affected the gait-related indices and the knee flexion, though the implication was greater for the individuals with PD than the healthy old. Our results indicated that the gait-related indices of individuals with PD showed a lack of dynamic balance [as evident from the correlation of gait-related indices with the FES score (see Section “Implication of Pathway With Varying Turns on Gait-Related Indices: For Task_C1_” and Section “Implication of Pathway With Varying Turns on Gait-Related Indices: For Task_C2_”)] when compared with age and gender-matched healthy old. A comparatively higher variation in knee flexion of individuals with PD than the healthy old was observed that might be indicative of their increased proneness to falls. Again, pathways with varying turn angles elicited variations in the counting performance under dual-task condition that was more prominent in the older group (with the counting performance error being more for the individuals PD than the healthy old and the discrepancy between the two groups being more for pathway with larger turn angle) than the younger group. Such an observation can be attributed to the prioritization of gait over cognition while employing the ‘posture first strategy’ by the healthy old and the ‘posture second strategy’ adopted by the individuals with PD during the dual task (attributed to the individuals dividing their attention between walking and counting while employing posture second strategy increasing their proneness to fall than that in the healthy old). In contrast, for the younger group, the implication of variation in the task condition on their gait-related indices was stronger than that due to pathways with varying turns.

Though the results were promising, our study had certain limitations. The sample size was limited. In the future, we plan to enroll a larger participant pool. Another limitation was the use of a limited variation in the pathway turns in our study. With daily living tasks requiring one to walk on pathways with turns up to 210° (de Morais Faria et al., 2016), we plan to investigate the implications of pathways with a turn angle >180° on one’s gait. Also, the walkway terrain was level ground. The implications might differ with different walkway terrains, such as uphill or downhill terrains. Another limitation of SmartWalk was that it looked to only one’s gait and knee flexion (for postural changes) for understanding one’s possible risk of fall. However, there can be other factors that can contribute to one’s risk of falls. For example, increased neck flexion, flexed posture, etc. (Yoshii et al., 2016) are also important elements contributing to postural changes that can have a role in one’s risk of falls. In the future, we plan to add these aspects to get a comprehensive view of one’s postural changes. Again, about the clinical measure for falls, we considered collecting FES scores from our participants. In the future, we plan to extend our study while incorporating other clinical measures, e.g., the Dynamic Gait Index (Badke et al., 2004) for quantifying one’s dynamic balance and fear of falls.

Notwithstanding the above limitations, our present study provided us with an understanding of the differentiated implications of pathways with varying turn angles and task conditions on at least some of the important aspects of one’s gait. Also, SmartWalk can offer valuable information to the clinicians regarding specific turn angles and task conditions that can be detrimental to individuals with PD about their falls, thereby offering pre-clinical inputs to the design of intervention studies.

## Data Availability Statement

The original contributions presented in the study are included in the article/supplementary material, further inquiries can be directed to the corresponding author.

## Ethics Statement

The studies involving human participants were reviewed and approved by Institutional Ethics Committee (IEC), IIT Gandhinagar. The patients/participants provided their written informed consent to participate in this study.

## Author Contributions

PP and UL drafted the manuscript and contributed to the experiment design, experimental data collection with healthy and individuals with Parkinson’s disease, data analysis, and statistical analysis. NJ and SD contributed to the enrolment of individuals with Parkinson’s disease and accessing their clinical measures. NP and MK contributed to the design of SmartWalk from the perspective of ergonomics and the pathway delineator. All authors read, corrected/commented on, and approved the final manuscript.

## Conflict of Interest

The authors declare that the research was conducted in the absence of any commercial or financial relationships that could be construed as a potential conflict of interest.

## Publisher’s Note

All claims expressed in this article are solely those of the authors and do not necessarily represent those of their affiliated organizations, or those of the publisher, the editors and the reviewers. Any product that may be evaluated in this article, or claim that may be made by its manufacturer, is not guaranteed or endorsed by the publisher.
